# Point-of-care HbA1c testing in a tertiary, university referral center: living up to the potential?

**DOI:** 10.1007/s12020-026-04615-6

**Published:** 2026-05-02

**Authors:** Lukas van Baal, Lynn Marlene Srasra, Annie Mathew, Theresia Sarabhai, Janina Kreuz, Roland Assert, Marc Wichert, Dagmar Führer, Denise Zwanziger

**Affiliations:** 1 Department of Endocrinology, Diabetes and Metabolism and Clinical Chemistry, Division of Laboratory Research, University Hospital Essen, University Duisburg-Essen, Essen, Germany; 2 Central Laboratory, University Hospital Essen, University Duisburg-Essen, Essen, Germany

**Keywords:** Diabetes, HbA1c, Point-of-care testing, Prediabetes, Real-world setting

## Abstract

**Background:**

Point-of-care (POC) HbA1c analysis offers an easy and rapid approach of assessing an individual’s glycemic status. However, its accuracy and diagnostic potential remain controversial. Here we compared POC vs. reference laboratory HbA1c testing in selected endocrine patient cohorts at a tertiary, university referral center using the widely established Afinion 2 analyzer.

**Methods:**

Single-center observational study of consecutive patients referred to the outpatient clinics of the Department of Endocrinology, Diabetes and Metabolism at the University Hospital Essen over 12 weeks. Venous blood samples for POC and laboratory HbA1c (Tosoh) testing were obtained simultaneously. Accuracy and diagnostic capacity of the POC HbA1c testing were evaluated.

**Results:**

Among 554 patients 47.1% (*n* = 261) were normoglycemic, 15.9% (*n* = 88) had prediabetes and 37.0% (*n* = 205) diabetes. Comparison of POC and laboratory HbA1c testing showed that 87.0% of POC values fell within ± 5.0% of the corresponding reference method values. When applying the German Medical Association guidelines, 58.7% of the POC values fell within ± 3.0% of the corresponding reference method values. ROC-analysis demonstrated an optimal cut-off for diagnosis of prediabetes of 5.65% (38 mmol/mol) and for diabetes of 6.25% (45 mmol/mol). Considering POC HbA1c testing as only tool for treatment decision, a medication error would have occurred in 12/195 patients (6.2%).

**Conclusion:**

Our study demonstrated, that POC HbA1c testing offers correlation but insufficient analytical agreement and clinically relevant misclassification compared to HPLC reference testing, highlighting that analytical validation in controlled environments does not automatically translate into diagnostic interchangeability in complex tertiary referral cohorts.

**Supplementary Information:**

The online version contains supplementary material available at 10.1007/s12020-026-04615-6.

## Introduction

 Diabetes and prediabetes are highly prevalent and associated with increased morbidity and mortality [1, 2]. Accurate detection of dysglycemia is therefore essential. Based on technological advances and validation studies the American Diabetes Association (ADA) supports POC HbA1c testing to facilitate rapid therapeutic adjustments and enhance outpatient diabetes management [3–5]. German regulation defined by the German Medical Association (GMA) permit POC testing under strict quality control but do not endorse replacement of standardized laboratory diagnostics [6]. The most widely used POC systems include Afinion (Alere Technologies AS, Waltham, MA, USA) and DCA Vantage (Siemens Medical Solutions Diagnostics, Tarrytown, NY, USA). Both systems are commercially available and routinely implemented in diabetological care in Germany.

However, most validation studies of POC HbA1c testing were conducted in controlled laboratory or primary care environments with relatively homogeneous populations [7–9]. Tertiary endocrine referral centers differ fundamentally. Patients often present with persistent dysglycemia, chronic kidney disease (CKD), anemia, or post-transplant status. Altered erythrocyte turnover, erythropoiesis-stimulating agents, iron deficiency, or carbamylation may influence HbA1c independently of glycemic status [10–13]. Thus, despite strong global evidence supporting POC HbA1c testing, analytical performance observed in validation settings may not translate to real-world highly specialized endocrine populations. Furthermore, in Germany laboratory HbA1c measurement is standardized, accredited, and reimbursed. In this context POC testing primarily aims to accelerate clinical workflow rather than overcome infrastructural limitations, where POC testing may primarily address limited access or cost constraints [14].

To address these gaps we evaluated the analytical accuracy and diagnostic validity of the Afinion 2 POC HbA1c analyzer in patients of a tertiary university endocrine outpatient department in Germany.

## Materals and methods

### Study design

This single-center observational study included consecutive adult patients attending endocrine outpatient clinics at the Department of Endocrinology, Diabetes and Metabolism, University Hospital Essen, between March and June 2021. No HbA1c-based inclusion or exclusion criteria were applied. There was no preselection regarding CKD, anemia, or other comorbidities, and no criteria were used to determine whether a patient underwent POC testing. All patients underwent simultaneous venous POC and laboratory HbA1c measurement from the same blood draw. No repeated sampling or measurement was conducted.

No formal a priori sample size calculation was performed. The primary objective was descriptive evaluation of analytical agreement and diagnostic performance under routine clinical conditions rather than hypothesis-driven interventional testing.

The study as well as the written informed consent documents adhered to the principles of the Declaration of Helsinki and were reviewed and approved by the institutional ethics committee. Clinical management was based exclusively on laboratory results.

### Description of patients

Patients were admitted to our department for follow-up of known diabetes (34.8%, *n* = 193), pituitary adenoma (34.5%, *n* = 191), for endocrine care after solid organ transplantation (14.1%, *n* = 78), and other endocrine disorders (16.6%, *n* = 92) (supplemental Fig. 1). The glycemic status of all patients was evaluated prior to biochemical testing using a standardized clinical assessment. This included a structured medical interview conducted by the treating physician regarding prior diagnosis of prediabetes or diabetes, review of the electronic health record (EHR), including documented diagnoses in discharge summaries and outpatient reports, review of previous laboratory results, and verification of current and past antidiabetic medication prescription. Patients undergoing metformin treatment were explicitly asked whether the medication was prescribed due to a current diagnosis of diabetes or as a preventive measure for prediabetes to avoid worsening glycemic status. No patient reported the latter.

**Fig. 1:**
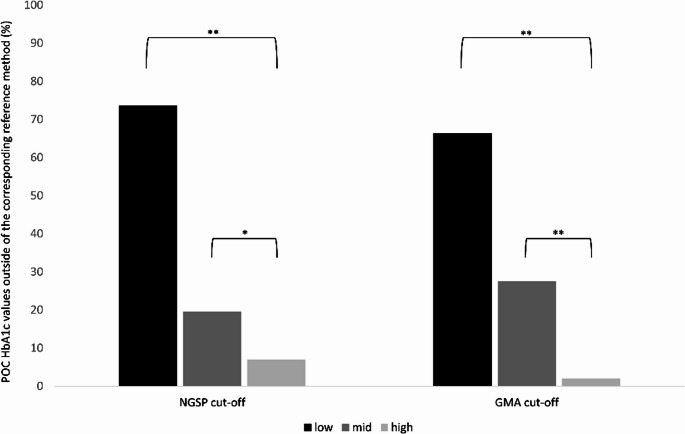
Percentage of POC HbA1c values outside the NGSP cut-off (n=72) and the GMA cut-off (n=229) of the corresponding reference method values across the glycemic range. POC, Point-of-care; NGSP; National Glycohemoglobin Standardization Program; GMA, German Medical Association. Black bar low HbA1c range (= 4.5-5.9%); dark grey bar, mid HbA1c range (6.0-7.5%), light grey bar, high HbA1c range (> 7.5%). *, p<.05; **, p<.01.

Demographic characteristics and biochemical results of glycemic status, chronic kidney disease (CKD, defined by glomerular filtration rate (GFR) following KDIGO criteria [15]) and overall hemoglobin (Hb) were obtained from the EHR. EHR was also reviewed for a documented red blood cell transfusion within the three months prior to HbA1c measurement. However, none of the included patients had received a documented red blood cell transfusion within three months prior to HbA1c measurement.

Biochemical glycemic status was defined according to the American Diabetes Association (ADA) Standards of Care based on HbA1c levels measured by the laboratory reference method [5]. D was defined as HbA1c ≥ 6.5% (47.5 mmol/mol). Prediabetes was defined as HbA1c 5.7–6.4% (38.8–46.5 mmol/mol). Patients with an HbA1c < 5.7% (38.8 mmol/mol) and absence of known history of prediabetes/diabetes were classified as normoglycemic.

### POC HbA1c testing

POC HbA1c testing was performed using the Afinion 2 analyzer (Alere Technologies AS, Waltham, MA, USA), a fully automated boronate affinity assay certified by the NGSP [16]. According to the manufacturer’s instructions for use, both capillary finger-prick blood and venous whole blood are approved sample types. In the present study, venous whole blood was used. Venous blood samples were obtained by routine phlebotomy and POC testing was performed without delay. All measurements were performed by trained medical staff who had received standardized instruction on device handling. Internal quality control procedures were carried out in accordance with the manufacturer’s specifications, including regular use of control materials and routine instrument calibration checks.

### Laboratory reference testing

HbA1c was determined from the same venous blood draw, in EDTA blood tubes with the Tosoh Automated Glycohemoglobin Analyzer HLC-723G8 (Tosoh Bioscience, Tokyo, Japan) by high performance liquid chromatography (HPLC) using a three-step salt gradient with a measuring range of 2.4% (3 mmol/mol) to 22.3% (220 mmol/mol). HbA1c testing in our clinical chemistry department is accredited according to DIN EN ISO 15189:2014 and NGSP [16].

### Statistical analysis

Data were analyzed using GraphPad Prism (GraphPad Software, Inc., San Diego, CA) and SPSS 27.0 (IBM Corporation, Armonk, NY). Continuous variables are presented as mean ± standard deviation (SD), and categorical variables as absolute numbers and percentages. All tests were two-sided, and a p-value < 0.05 was considered statistically significant.

Prior to parametric testing, continuous variables were assessed for normal distribution using visual inspection of histograms and Q–Q plots, and homogeneity of variances was evaluated using Levene’s test. When assumptions were met, parametric methods were applied. For descriptive comparisons of baseline characteristics, patients were grouped according to glycemic status based on laboratory HbA1c (normoglycemic, prediabetes, diabetes), and predefined HbA1c reference ranges (low: 4.5–5.9%; mid: 6.0–7.5%; high: >7.5%).

To compare demographic and biochemical variables one-way ANOVA was applied for continuous variables. Bonferroni correction was used for post hoc pairwise comparisons to control for type I error inflation. To assess whether CKD and anemia independently influenced HbA1c bias or diagnostic classification, analysis of covariance (ANCOVA) models were performed. In these models age was included as a covariate and sex, CKD (yes/no), and anemia (yes/no) were included as fixed between-subject factors.

Agreement between POC HbA1c and HPLC reference measurements was evaluated using Kendall’s Tau correlation coefficient to assess monotonic association between methods. Correlation analysis was not interpreted as agreement but as a measure of association. Method comparison was performed using Passing–Bablok regression to assess systematic and proportional bias, supplemented by Deming regression (λ = 1) to account for measurement error in both methods. Slope and intercept with 95% confidence intervals (CI) were reported. Deviation of slope from 1 and intercept from 0 was interpreted as proportional or constant bias, respectively. Bland–Altman analysis was conducted to quantify mean bias and limits of agreement (± 1.96 SD) [17].

Analytical performance was further assessed by calculating the proportion of POC values within ± 5% (NGSP criteria) and ± 3% (GMA criteria) of the reference method. Predefined subgroup analyses were conducted for HbA1c ranges (4.5–5.9%; 6.0–7.5%; >7.5%), anemia (yes/no) and CKD (yes/no).

For evaluation of diagnostic performance in patients without known dysglycemia, sensitivity, specificity, positive likelihood ratio (LR+), and negative likelihood ratio (LR−) were calculated with 95% confidence intervals. Receiver operating characteristic (ROC) analysis was performed to determine optimal POC HbA1c cut-offs for identifying prediabetes and diabetes, and area under the curve (AUC) values with 95% confidence intervals were reported. Exploratory optimal cut-offs were determined using the Youden index (J = sensitivity + specificity − 1). Treatment threshold assumptions were based on ADA Standards of Care for HbA1c targets [18].

## Results

### Demographic characteristics

A total of 554 patients (54.2% [*n* = 300] male) with a mean age of 51.7 ± 15.7 years, mean BMI of 28.0 ± 6.5 kg/m² and mean HbA1c value (laboratory testing) of 6.0 ± 1.1% were included (Table 1).

**Table 1:** Demographic characteristics of the study population. The data are broken down by the entire study cohort and by glycemic status. Additionally, individual variables were compared according to glycemic status with ^a^ = normoglycemic vs. prediabetes; ^b^ = normoglycemic vs. diabetes and ^c^ = prediabetes vs. diabetes. Data are presented as mean + standard deviation or absolute numbers and percentage obtained

	All	Range	Normoglycemic	Prediabetes	Diabetes
*N*	554	-	261/554 (47.1%)	98 /554 (17.7%)	195/554 (35.2%)
History of dysglycemia	215/554 (38.8%)	-	-	30/98 (30.6%)	185/195 (94.9%)
Newly diagnosed	78/554 (14.1%)	-	-	68/98 (69.4%)	10/195 (5.1%)
Men	300/554 (54.2%)	-	154/300 (51.3%)	44/300 (14.7%)	102/300 (34.0%)
Women	254/554 (45.8%)	-	107/254 (42.1%)	44/254 (17.3%)	103/254 (40.6%)
Age (years)	51.7 (± 15.7)	19–81	48.0 (± 15.5)^b, c***^	56.2 (± 14.8)^a***^	54.6 (± 15.2)^a***^
BMI (kg/m^2^)	28.0 (± 6.5)	18.7–41.1	28.2 (± 6.8)	27.2 (± 5.8)	28.1 (± 6.5)
Hemoglobin (g/dl)	13.4 (± 1.9)	5.8–18.9	13.5 (± 1.9)	13.3 (± 1.8)	13.2 (± 1.9)
Anemia (Hb < 12.0 g/dl)	71/554 (12.8%)	-	25/261 (9.6%)	16/98 (16.3%)	30/195 (15.4%)
CKD KDIGO	107/554 (19.3%)	-	51/261 (19.5%)	17/98 (17.3%)	39/195 (20.0%)
HPLC HbA1c (%)	6.0 (± 1.1)	4.4–9.2	5.3 (± 0.3)^a, b***^	5.9 (± 0.2)^a, c***^	6.9 (± 1.0)^b, c***^
POC HbA1c (%)	5.9 (± 1.0)	4.5-9.0	5.2 (± 0.4)^a, b***^	5.7 (± 0.3)^a, c***^	6.9 (± 1.0)^b, c***^

N, number; BMI, body mass index; kg, kilogram; m^2^, square meters; g, gram; dl, deciliter; Hb, hemoglobin; GFR, glomerulation filtration rate; CKD, chronic kidney disease; KDIGO, Kidney Disease: Improving Global Outcomes; POC, point-of-care, ***, *p*<.001

History of dysglycemia was noted in 215 patients, of these 185 with known diabetes (type 1 4.9% [*n* = 9], type 2 67.0% [*n* = 124] and new-onset diabetes after organ transplantation 28.1% [*n* = 52]). Mean HbA1c values were 5.3 ± 0.3% for normoglycemic, 5.9 ± 0.2% for prediabetes and 6.9 ± 1.0% for diabetes. CKD was present in 107 (19.3%) and anemia in 71 patients (12.8%).

### Accuracy of POC HbA1c

POC HbA1c results ranged from 4.5 to 9.0% and results of the reference method from 4.4 to 9.2%. POC HbA1c values were highly correlated with the reference method HbA1c values with a Kendell`s τ coefficient of 0.858. Passing Bablok regression yielded a slope of 1.000 (95%CI: 1.000–1.000). Deming regression (λ = 1) produced a slope of 1.022 (95% CI 1.002–1.044) and intercept of -0.23 (95% CI -0.38 - -0.10).

The absolute bias between POC and reference method HbA1c values was 0.08 ± 0.3% and the relative bias.

-1.3 ± 4.4%, with no significant differences across the HbA1c range (Table 2). Applying NGSP recommendations, 87.0% of the POC values fell within ± 5.0% of the corresponding reference method values. Regarding the guidelines of the GMA, 58.7% of the POC values fell within ± 3.0% of the corresponding reference method values. Percentage of values outside ± 5.0% (*n* = 72), as well as ± 3.0% (*n* = 229) of the corresponding reference method values increased significantly from the high to the low HbA1c range (NGSP cut-off: high: 6.9% (*n* = 5), mid: 19.5% (*n* = 14), low: 73.6% (*n* = 53), high vs. mid p.05, mid vs. low *p*<.01 and GMA cut-off: high: 6.1% (*n* = 14), mid: 27.5% (*n* = 63), low: 66.4% (*n* = 152), high vs. mid *p*<.01, mid vs. low *p*<.01; Fig. 1). Detailed information about HbA1c distribution and accuracy across the glycemic range is provided in Table 2.

**Table 2:** POC HbA1c testing components of bias. The data is organized in two ways: according to the HbA1c range in the left-hand column and according to the type of bias in the top row. Data are presented as mean *±* standard deviation or absolute numbers and percentage obtained

HbA1c range	*N*	Mean HbA1c	Relative Bias %	Absolute Bias	*≤* ±5.0% of the corresponding reference method values	*≤* ±3.0% of the corresponding reference method values
Overall	554	6.0% (± 1.1)	-1.30% (± 4.4)	0.08 (± 0.3)	482/554 (87.0%)	325/554 (58.7%)
Low (4.5–5.9%)	360/554 (65.0%)	5.4% (± 0.4)	-1.30% (± 4.9)	0.08 (± 0.3)	307/360 (85.3%)	208/360 (57.8%)
Mid (6.0-7.5%)	155/554 (28.0%)	6.4% (± 0.3)	-1.60% (± 3.2)	0.10 (± 0.2)	141/155 (91.0%)	92/155 (59.4%)
High (> 7.5%)	39/554 (7.0%)	8.0% (± 1.2)	-1.20% (± 2.9)	0.09 (± 0.2)	34/39 (87.2%)	25/39 (64.1%)

N, number

Subgroup analysis of patients with and without anemia and chronic kidney disease revealed no significant differences: In patients with anemia the absolute bias was 0.03 ± 0.3% (p.19) and relative bias was.

-0.6 ± 6.0% (p.23). Regarding NGSP recommendations 87.3% (*n* = 62; p.94) of the POC values fell within ± 5.0% of the corresponding reference method values and regarding the GMA guidelines 63.4% (*n* = 45; p.37) of the POC values fell within ± 3.0% of the corresponding reference method values. In patients with CKD the absolute bias was 0.1 ± 0.3% (p.53) and the relative bias was − 0.8 ± 4.7% (p.29). NGSP recommendations were fulfilled in 83.2% (*n* = 89; p.29) and GMA guidelines in 59.8% (*n* = 64; p.83) of POC values.

**Fig. 2:**
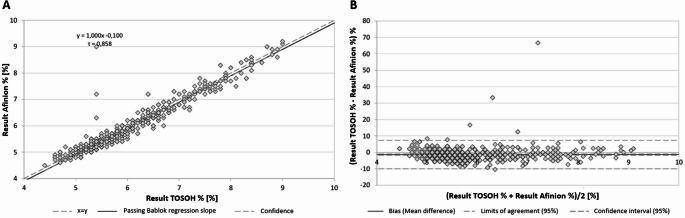
Concordance between Afinion POC and HPLC (TOSOH) HbA1c testing. A: Passing Bablok regression. B: Bland-Altman-Analysis. A: Passing Bablok regression. B: Bland-Altman-Analysis

BAA revealed a mean relative bias of -1.3% (95%CI: -1.7-1.0%), indicating the presence of significant systemic bias within the POC testing (Fig. 2). The distance between the 95% limits of agreement and the range in which the actual deviation would lie with a 95% probability was − 10.0-7.3% (range = 17.3%).

### Diagnostic performance

**Fig. 3:**
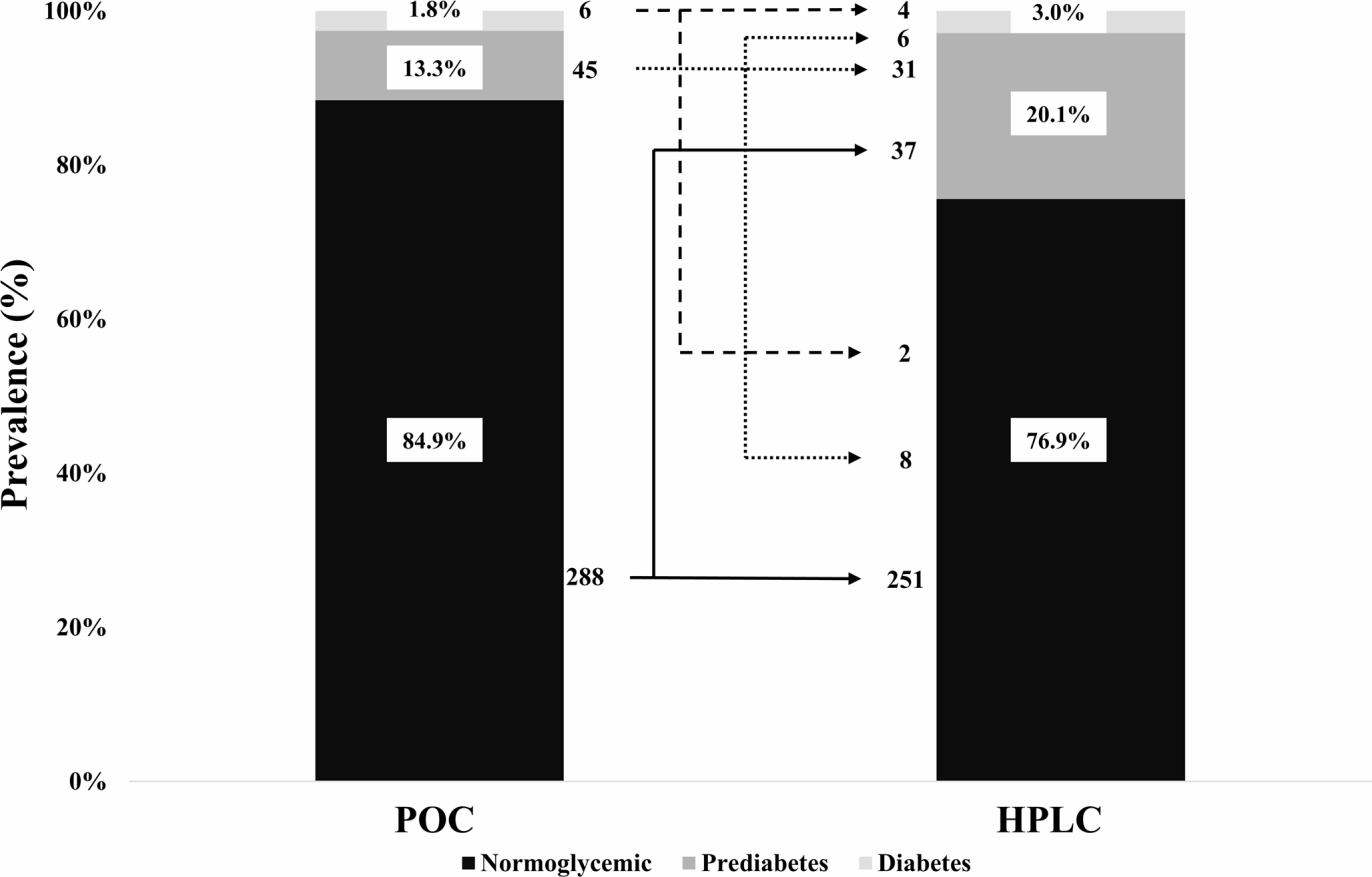
Prevalence of new-onset dysglycemia in patients admitted to the outpatient care using two different HbA1c testing methods (POC vs. HPLC). Point-of-care (POC), screening for dysglycemia with POC HbA1c testing; HPLC, Screening for dysglycemia with high-performance liquid chromatography (HPLC) HbA1c testing; black panel, normoglycemic; dark grey panel, prediabetes; light grey panel, diabetes; Data are presented as percentage obtained in the bars and in absolute numbers beside the bars with arrows indicating change in patients' glycemic status due to choice of diagnostic strategy with continuous black line for normoglycemic patients, dotted black line for patients with prediabetes and dashed black line for patients with diabetes in the POC Hba1c testing.

Diagnostic performance analyses were restricted to hitherto undiagnosed patients (*n* = 339). Within this subgroup, newly diagnosed diabetes was present in ten patients and newly diagnosed prediabetes in 68 patients, resulting in comparatively small event numbers for sensitivity estimation. If POC HbA1c testing was considered as screening tool for diagnosis of dysglycemia, prevalence of prediabetes was 13.3% (*n* = 45) and of diabetes was 1.8% (*n* = 6) in hitherto undiagnosed cases. Thirty-seven patients with prediabetes would have been classified as normoglycemic. Conversely, eight patients with normoglycemia would have been diagnosed with prediabetes as well as two patients with diabetes. Six patients with diabetes would have been misclassified as prediabetes (Fig. 3).

Subgroup analyses revealed the following: In hitherto undiagnosed cases, misclassification of dysglycemia occurred in 21.6% of patients with anemia (8/37) compared to 13.2% in patients without anemia (*p*=.17). In patients with CKD, misclassification occurred in 21.9% (14/64) compared to 13.2% in patients without CKD (*p*=.05).

**Fig. 4:**
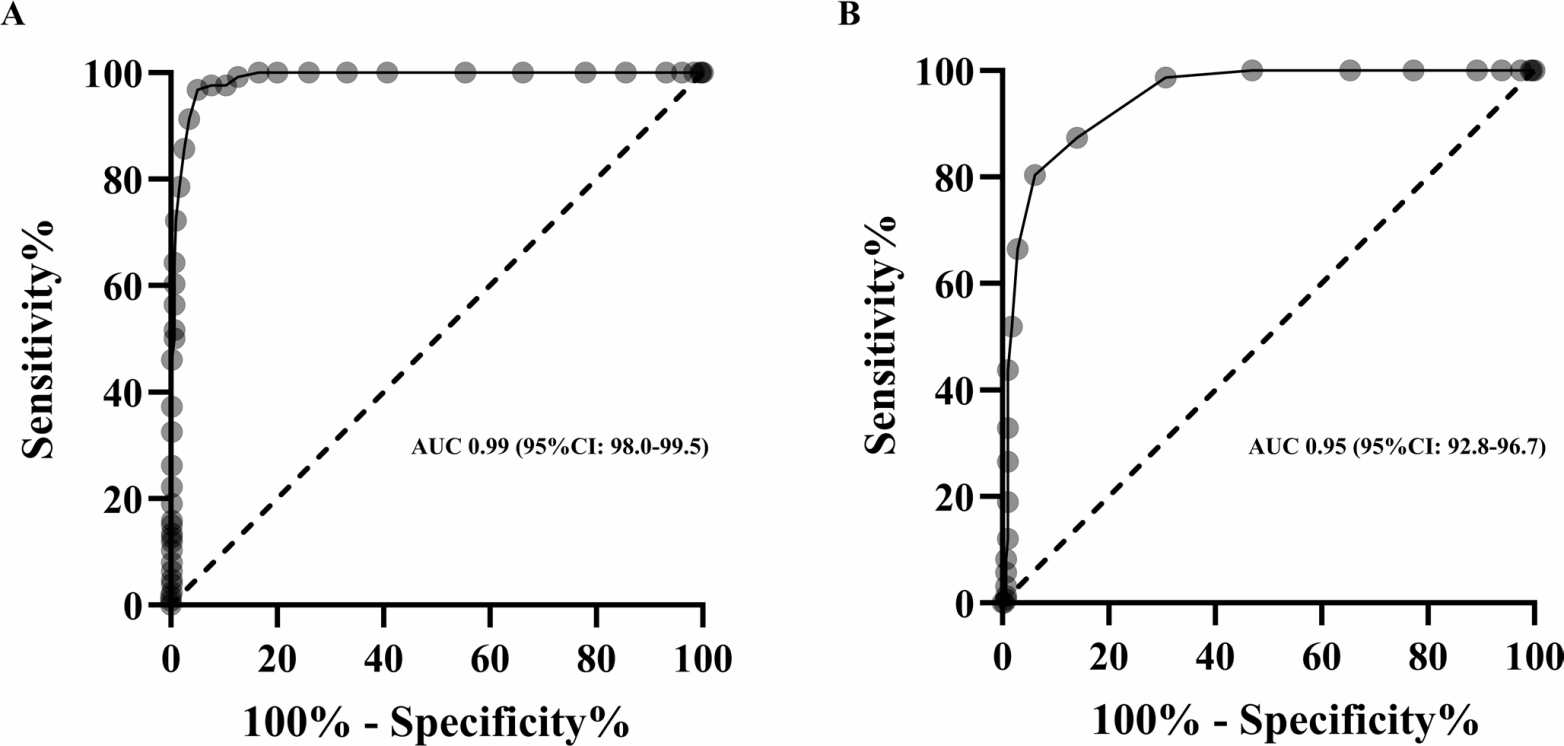
Receiver operating characteristic (ROC) curve for POC HbA1c to identify the presence of undiagnosed diabetes (A) and prediabetes (B); AUC, area under the curve; 95%CI, 95% confidence interval.

If ADA criteria [19] for diagnosis of dysglycemia was applied, POC HbA1c testing showed a sensitivity of 35.1% (95%CI: 2.4–47.1) and a specificity of 98.1% (95%CI: 95.6–99.4) with a LR + of 18.2 (95%CI: 7.2–45.7) and a LR- of 0.7 (95%CI: 0.6–0.8) for diagnosis of prediabetes. For diagnosis of diabetes a sensitivity of 70.0% (95%CI: 34.8–93.3) and a specificity of 99.2% (95%CI: 97.2–99.9) was calculated, with a LR + of 89.6 (95%CI: 21.3-377.8) and a LR- of 0.3 (95%CI: 0.1–0.8). ROC-analysis revealed an optimal cut-off for diagnosis of prediabetes of 5.65% (sensitivity: 80.4%, specificity: 93.9%, AUC 0.95, 95%CI: 0.93–0.97) and for diabetes of 6.25% (sensitivity: 96.8%, specificity: 94.9%, AUC 0.99, 95%CI: 0.98–0.99) (Fig. 4).

### Influence on treatment decision

If the POC HbA1c testing rather than laboratory measurement had been used to make treatment decisions for patients with a known diagnosis of diabetes, our results show that treatment decisions would have been different for 12 out of 195 patients (6.2%). In eight of these patients, an indicated intensification of antidiabetic treatment would have been omitted and in two patients, an intensification of antidiabetic treatment would have been carried out incorrectly. In two other patients, antidiabetic treatment would have been initiated despite the absence of diabetes.

## Discussion

### Impaired accuracy of POC versus laboratory HbA1c testing

The interpretation of diagnostic performance in our study must be contextualized within the referral structure of a tertiary endocrine center. Therefore, our study simultaneously evaluates two clinically distinct scenarios: (1) detection of previously unrecognized dysglycemia and (2) treatment-guiding accuracy in patients with known diabetes. This dual perspective reflects real-world practice in tertiary care rather than classical screening validation. In this tertiary endocrine referral cohort, POC HbA1c testing using the Afinion 2 analyzer showed strong correlation with HPLC-based laboratory reference testing, but failed to meet predefined analytical agreement criteria. This distinction is clinically relevant. Correlation reflects association, whereas interchangeability requires narrow limits of agreement and compliance with established quality standards. According to NGSP criteria, ≥ 90% of POC results should fall within ± 5% of a certified laboratory method [16], and the German Medical Association (GMA) defines a stricter ± 3% limit [6]. In our cohort, these analytical thresholds were not fulfilled.

Importantly, in our study, venous blood was used for both POC and laboratory testing from the same blood draw. This design minimized preanalytical variability and eliminated capillary sampling bias due to inconsistent sample volume, interstitial fluid dilution, or operator dependency [9, 20]. The use of venous samples under standardized conditions therefore strengthens internal validity and indicates that the observed discrepancies reflect analytical performance rather than sampling artifacts. It is conceivable that real-world performance using capillary blood may be equal or potentially even more variable. However, earlier validation studies of the Afinion platform reported excellent concordance with NGSP requirements [7, 8]. In the multicenter study by Sobolesky et al. [7], the analytical performance of the Afinion AS100 was evaluated across several laboratory sites using venous samples with an evenly distributed HbA1c range extending well into higher glycemic values. Similarly, Arnold et al. [8] conducted a laboratory-based accuracy and precision assessment comparing the Afinion AS100 with NGSP-certified reference methods under standardized preanalytical and analytical conditions. In contrast, our investigation reflects real-world clinical implementation in a tertiary referral center, characterized by a predominance of lower HbA1c values. The predominance of values in the lower glycemic range is relevant, as analytical variability may have greater proportional impact near diagnostic cut-offs. Furthermore, our population presented high prevalence of complex comorbidities such as CKD and anemia. CKD and anemia are common in tertiary endocrine cohorts and are known to influence HbA1c through altered erythrocyte turnover, iron metabolism, and carbamylation [11–13]. In our study, subgroup analyses demonstrated that misclassification rates were numerically higher in CKD and anemia. While these differences were not statistically significant for anemia and only borderline for CKD (*p* = .05), they suggest increased vulnerability in these subgroups and that CKD and anemia likely contributed to the observed systemic negative bias. However, these analyses were not powered to detect moderate effects. Importantly, the negative bias identified in Bland–Altman analysis was present across the entire cohort and not confined to these subgroups, indicating that impaired diagnostic performance reflects a broader analytical variability in this tertiary referral population rather than being exclusively driven by CKD or anemia.

### POC HbA1c testing can support and accelerate treatment decisions in patients at HbA1c values > 7.5%

The clinical implications of impaired agreement depend on intended application. In our setting of a tertiary endocrine referral center, two distinct scenarios are relevant: detection of previously unrecognized dysglycemia and therapeutic monitoring in patients with established diabetes. For example, missing a diagnosis of prediabetes or diabetes can delay structured follow-up and preventive interventions, resulting in significantly impaired outcomes for these already multimorbid patients [21]. These factors may not apply, or may apply less strongly, to HbA1c POC testing in most other settings. We found that, four patients with newly diagnosis of diabetes in laboratory HbA1c testing would have been misclassified as prediabetes and 37 patients with prediabetes as normoglycemic with POC HbA1c testing.

For screening purposes, diagnostic sensitivity is critical in a prevalent disease with effective preventive strategies [22]. In hitherto undiagnosed individuals, ROC-analysis with application of ADA HbA1c diagnostic thresholds to POC HbA1c values resulted in an optimal cut-off for diagnosing prediabetes of 5.65% (38 mmol/mol) with limited sensitivity (80.4%). For diagnosis of diabetes the optimal cutoff was 6.25% (45 mmol/mol), with a very good sensitivity of 96.8%. Specificity for both cut-offs was high (93.9% and 94.9%, respectively). The only other study with an ROC analysis for POC HbA1c testing was conducted by Pi et al. with a Sinocare (China) analyzer [23]. They provided a cut-off for prediabetes diagnosis of 5.25% ((34 mmol/mol), sensitivity: 89.5%; specificity: 77.4%) and for diabetes of 5.95% ((42 mmol/mol), sensitivity: 88.2%; specificity: 88.1%). The difference may be due to the ethnicity and age of the population studied by Pi et al. (63.0 ± 15.0. vs. 51.7 ± 15.7 years in our study), resulting in a higher prevalence of prediabetes and diabetes [24]. Importantly, our study was not designed as a formal diagnostic accuracy trial with predefined power calculations for sensitivity thresholds. but rather as analytical characterization of diagnostic discrimination. Therefore, these exploratory cut-offs were intended to illustrate how far the device would need to deviate from ADA thresholds [19] to improve sensitivity in a tertiary care environment. Consequently, our thresholds were derived exploratorily from a subgroup with limited event numbers (ten newly diagnosed diabetes cases) and should be interpreted as evidence of clinically relevant discrepancy under routine conditions rather than as replacing of standardized laboratory diagnostics for population-wide screening strategies.

Although the ADA has recently recommended POC HbA1c testing for the rapid adjustment of antidiabetic treatment, studies comparing its therapeutic potential to that of standard laboratory testing are scarce and have not been designed for specific endocrine cohorts [25, 26]. However, HbA1c remains a central parameter for long-term glycemic target evaluation and treatment adjustment, typically aiming for < 7.5% in most non-pregnant adults, with individualized targets depending on age, comorbidity, and hypoglycemia risk. While insulin intensification decisions are clinically driven by glucose profiles, HbA1c serves as a validated integrative marker of chronic glycemic exposure [18]. Our analysis therefore reflects a guideline-based threshold approach rather than real-time glucose-driven insulin titration. In our patients with established diabetes, reliance solely on POC HbA1c values would have altered therapeutic decisions in 6.2% of cases. Most discrepancies involved omission of indicated treatment intensification, though inappropriate initiation and overtreatment were also observed. In a multimorbid tertiary cohort, both delayed intensification and overtreatment are clinically relevant. Sustained hyperglycemia is associated with adverse outcomes [2, 27], whereas unnecessary intensification increases the risk of hypoglycemia [28]. Although, the absolute number of affected individuals was modest, these findings suggest a potential vulnerability of POC HbA1c testing in metabolically complex patients and underscore that even small systematic deviations may influence management in high-risk populations.

Importantly, interpretation of our findings must consider the healthcare context. In Germany, laboratory HbA1c measurement is standardized, accredited (DIN EN ISO 15189) and widely accessible. In this environment, POC testing primarily aims to facilitate workflow and accelerate clinical decision-making rather than to overcome infrastructural barriers [14]. When reference testing is readily available and cost is not prohibitive, analytical equivalence becomes a prerequisite for standalone diagnostic use. Our data do not support such equivalence of POC testing demonstrating that analytical validation performance does not necessarily translate into reliable real-world diagnostic interchangeability in complex tertiary populations. In summary our results suggest, that POC HbA1c testing with the Afinion 2 analyzer may be more suitable for rapid assessment in clearly hyperglycemic patients, where minor deviations are unlikely to alter management. Conversely, values close to diagnostic or therapeutic HbA1c cut-offs of the ADA require higher analytical precision and laboratory confirmation.

### Limitations and strengths of the present study

The limitations of our study design should be acknowledged. Patients with HbA1c values > 9.0% were underrepresented. Consequently, device performance in patients with marked metabolic decompensation remains unassessed and limits conclusions in severe hyperglycemia. Our finding of an identical 95% confidence interval for the slope (1.000–1.000) elucidates this limitation. This interval is not due to a computational or reporting error but is a known property of the Passing–Bablok algorithm when applied to datasets with low variability or many identical measurement pairs. Another limitation is the absence of a formal a priori sample size calculation and the restricted statistical power of subgroup analyses. Consequently, subgroup sizes, particularly in newly diagnosed patients, may have been insufficient to detect moderate but clinically relevant differences in POC performance. Therefore, the absence of statistically significant subgroup effects should not be interpreted as proof of absence of biological influence. However, the relatively large number of paired measurements provided narrow confidence intervals for agreement and bias estimates, supporting the precision of the primary findings. Future, larger studies with predefined hypothesis-driven power calculations for high-risk comorbid populations may further strengthen external validation. Furthermore, the single-center design may limit generalizability to primary care settings with different patient characteristics. However, this tertiary referral environment was intentionally selected to evaluate performance under clinically demanding conditions, where diagnostic precision is particularly relevant. Finally, it should be noted, that carbamylation and hemoglobin variants were not systematically assessed, even though they are known to interfere with POC HbA1c testing [29]. Although none of our patients had a known hemoglobinopathy, heterozygous variants are common and may additionally contribute to variability of our results [30].

Strengths of the study include the relatively large number of paired measurements, simultaneous venous sampling for both POC and laboratory HbA1c testing and strict adherence to manufacturer specifications. This minimized preanalytical variation. This is particularly relevant, as a previous study revealed that POC testing carried out significantly later than laboratory testing results in significantly lower POC HbA1c values [31]. Moreover, to our knowledge, this is the first study assessing analytical performance and clinical implications of the Afinion 2 analyzer in a German tertiary referral setting. Therefore, our study population, predominantly comprising of Caucasian patients, differ substantially from previously studied primary care or multiethnic validation cohorts in controlled settings [32]. On the contrary, our study specifically addresses whether these findings translate to a tertiary university referral center and underscores the importance of context-specific validation in complex clinical environments.

## Conclusion

In this tertiary referral cohort, POC HbA1c testing demonstrated high correlation but insufficient analytical agreement and clinically relevant misclassification compared to HPLC reference testing, highlighting that analytical validation in controlled environments does not automatically translate into diagnostic interchangeability in complex real-world clinical settings. While it may support rapid therapeutic decisions in patients with clearly elevated HbA1c values, laboratory confirmation remains necessary when results approach diagnostic or treatment-defining thresholds.

## Supplementary Information

Below is the link to the electronic supplementary material.

Supplementary Material 1

## Data Availability

Individual participant data from this study will be available in an anonymized form from the publication date of this manuscript for the consecutive 24 months, on a collaborative basis for individual participant data meta-analyses. Proposals should be directed to Lukas van Baal (Lukas.van-Baal@uk-essen.de)
