# Supplementary figures and images for: Point-of-care HbA1c testing in a tertiary, university referral center: living up to the potential?
Source: Endocrine. 2026 May 2;91(1):160. doi: 10.1007/s12020-026-04615-6 (PMC13135566; doi:10.1007/s12020-026-04615-6)

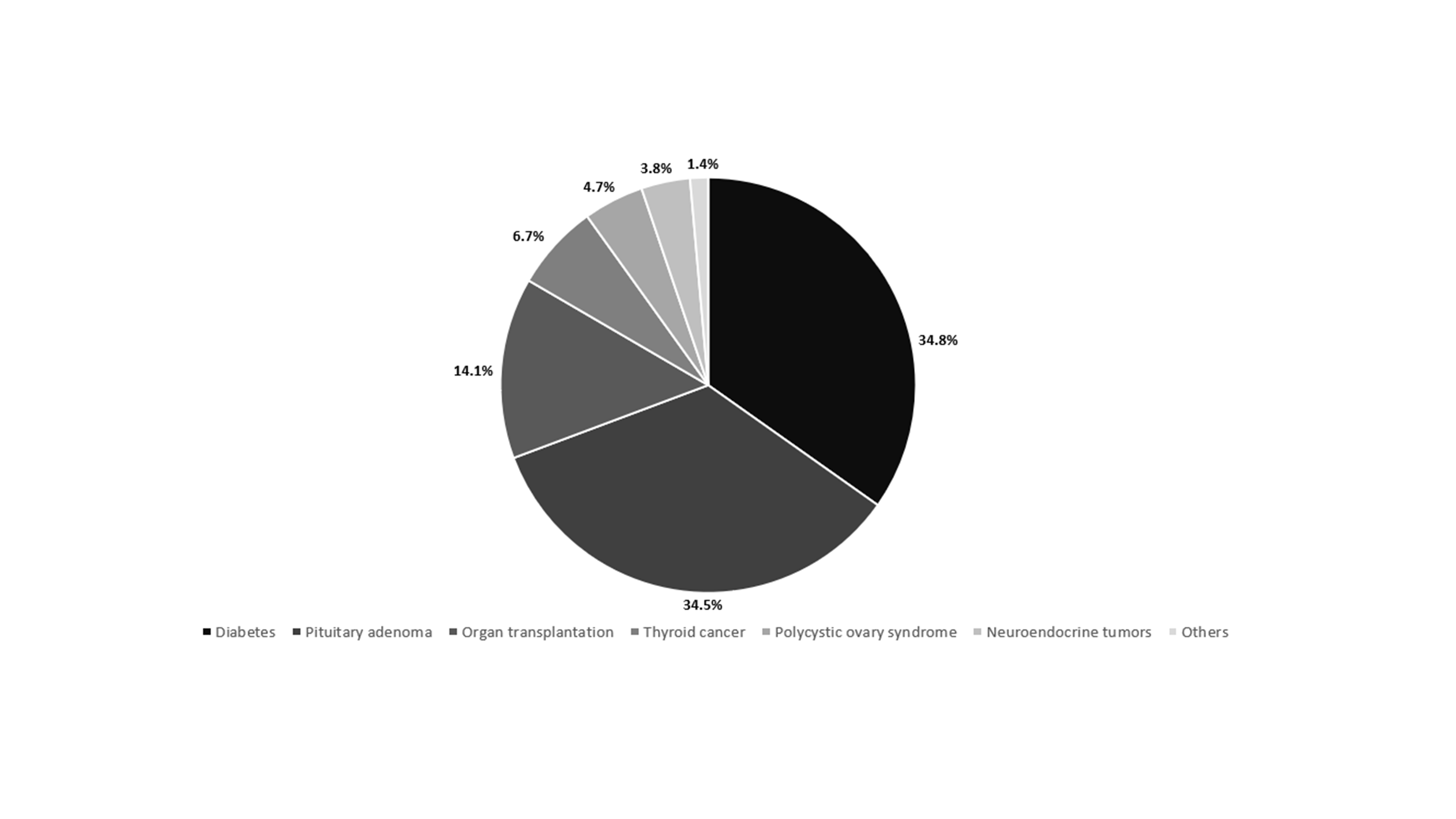

Supplement: Supplementary file 1 — Supplementary Material 1 [file 12020_2026_4615_MOESM1_ESM.tif]
